# LZAP promotes the proliferation and invasiveness of cervical carcinoma cells by targeting AKT and EMT

**DOI:** 10.7150/jca.39359

**Published:** 2020-01-14

**Authors:** Yi-Fang Dai, Na Lin, De-Qin He, Mu Xu, Li-Ying Zhong, Shu-Qiong He, Dan-Hua Guo, Ying Li, Hai-Long Huang, Xiang-Qing Zheng, Liang-Pu Xu

**Affiliations:** 1 Center of Prenatal Diagnosis, Fujian Provincial Maternity and Children's Hospital, Affiliated Hospital of Fujian Medical University, Fuzhou 350001, China; 2 Fujian Provincial Key Laboratory for Prenatal diagnosis and Birth Defect, Fuzhou 350001, China; 3 Department of Gynecology, Fujian Provincial Maternity and Children's Hospital, Fuzhou 350001, China

**Keywords:** LZAP, EMT, Cervical Carcinoma, Metastasis

## Abstract

**Objective**: To explore the relationship and mechanism of LZAP in the occurrence and development of cervical cancer and to provide a new target and intervention method for the treatment of cervical cancer.

**Methods**: Data mining and analysis of LZAP expression levels were performed using several online databases, including The Cancer Genome Atlas (TCGA). A cervical cancer cell line that stably overexpresses LZAP was established, and the effect of LZAP overexpression on cell proliferation, invasion, migration and tumor formation in vivo as well as its mechanism were explored.

**Results**: Our study shows that the expression of LZAP is upregulated in cervical cancer. The overexpression of LZAP can significantly promote the proliferation, colony formation, and invasion and migration abilities of cervical cancer cells. The tumorigenesis test in nude mice showed that overexpression of LZAP could promote the tumorigenicity of cervical cancer cells in vivo. LZAP could also promote the phosphorylation of AKT at position 473 and the epithelial-mesenchymal transition (EMT).

**Conclusion**: The expression of LAZP is increased in cervical cancer, which can enhance the invasion, metastasis, and EMT in cervical cancer cells by promoting AKT phosphorylation.

## Introduction

Cervical cancer is the third most common malignant tumor of the female reproductive tract and the fourth largest cause of cancer-related death worldwide 1, 2. Most patients already have progressive disease at the time of treatment, and many treatment methods have not achieved satisfactory results 3, 4. Presently, the mechanisms of proliferation, invasiveness and metastasis of cervical cancer have not been fully clarified 5. Moreover, specific and efficient intervention is lacking. Therefore, studies on the mechanism of the occurrence and development of cervical cancer can provide a new approach to determine targeted treatment of cervical cancer with important theoretical and clinical significance.

Cyclin-dependent kinase 5 activating binding protein (CDK5RAP3, also known as LZAP) and C48 and C42 were first identified as cyclin-dependent kinase 5 activating proteins p35 and p39 binding proteins, which are widely expressed in various tissues and cells 6, 7. In recent years, an increasing number of studies has shown that LZAP plays unique roles in different tumor types and participates in many classical tumors signaling pathways 8, 9. High expression of LZAP in cervical cancer has been reported in public databases, which suggests that LZAP may be involved in the occurrence and development of cervical cancer. However, no reports have been published on the role and mechanism of the LZAP gene in cervical cancer. In this study, we investigated the expression of LZAP in cervical cancer and paracancerous tissues as well as the role of LZAP in cervical cancer cells. We hope to provide a new gene target for the diagnosis and treatment of cervical cancer.

## Materials and Methods

### Immunohistochemistry

Paraffin blocks containing enough formalin-fixed tumor specimens were sliced continuously at a thickness of 4 μm and placed on silane-coated slides for immunohistochemical analysis. The slices were deparaffinized in xylene and rehydrated in 100, 100, 95, 85 and 75% ethanol solutions. Antigen retrieval was performed in 0.01 mol/l sodium citrate buffer (121 ℃ autoclaved 2 min, pH 6.0). Endogenous peroxidase activity was quenched by incubating the sections in 3% H_2_O_2_ at room temperature for 10 min. Then, the sections were washed in phosphate-buffered saline (PBS) and blocked with 10% goat serum (Zhongshan Biotechnology Co., Ltd.) for 30 minutes after which the slides were incubated with a rabbit anti-human LZAP (Abcam, Cambridge, MA, USA) antibody in a humidifying chamber overnight at 4 ℃. After the slides were washed again three times in PBS, the slides were incubated with the secondary antibody conjugated to horseradish peroxidase at room temperature for 30 minutes. The signal was developed with 3,3'-diaminobenzidine (DAB) solution and counterstained in 20% hematoxylin. Finally, all slides were dehydrated and mounted with a cover slip. For the negative control, antibody diluent was used in place of the primary antibody.

### Cell culture

Two strains of cervical cancer cells (HeLa and HCC94) were purchased from the Institute of Biochemistry and Cell Biology, Chinese Academy of Sciences (Shanghai, China). These cell lines were cultured in RPMI 1640 (Gibco, Grand Island, NY, USA) and DMEM/F12 1:1 medium each containing 10% fetal bovine serum (FBS) (Gibco, Grand Island, NY, USA) in an incubator with 5% CO2 at 37 ℃.

### Construction of stable cell lines

LZAP (nm_001278198)-overexpressing cell lines were established using lentivirus, and negative control (NC) cells were established using its corresponding empty vector lenti-emp. The day before lentivirus transfer, HeLa and HCC94 cells were inoculated in 6-well plates at a density of 5 × 10 ^ 4 cells/well (20 ≤ 30% coincidence degree). Polyphenylene (10 mg/ml) and Enhanced Infection Solution (GeneChem, China) were used to transfer lenti-LZAP into cells under suitable conditions. The corresponding empty vector (lenti-NC) was introduced into other cells at the same time by the same method. The cells were cultured in an incubator containing 5% CO_2_ at 37 ℃ for 6 h at which time the medium was replaced. To establish a stable cell line, 48 hours after the medium was replaced, the cells were screened in media containing puromycin (1 ≤ 2 mg ·ml, Sigma) for at least 1 week after transfection. The cells were harvested for mRNA, protein analysis and other detection methods. The LZAP sequence designed and synthesized is: forward 5'GCTGGTGGACAGAAGGCACT3'; reverse 5' TGTCCTGGATGGCAGCATTGA 3'.

### Cell proliferation assay

A 96-well plate was used to inoculate cervical cancer cells at a density of 1000 cells per well (100 μl cell suspension), and 100 μl of serum-free medium containing 10% CCK8 reagent was added at d0, d1, d2, and d3. Next, the OD450 was measured after 2 hours, and proliferation curves were generated. There were 5 replicate wells per cell type, and each experiment was repeated 3 times.

### Colony formation assay

Cervical cancer cells in the logarithmic growth phase were inoculated into a six-well plate at a density of 1000 cells/well. Cells were routinely cultured for 10 days, washed 3 times in phosphate-buffered saline (1×PBS), soaked in 70% methanol for 15 min, and stained with crystal violet. After 30 min, they were rinsed 3 times in PBS (note that the cells should not be washed away after each wash), and the number of colonies was counted by light microscopy. Each experiment was repeated 3 times.

### Cell invasion and migration assay

In all, 2×10^4 cells (100 μl cell suspension) were added to Transwell chambers, after which 500 μl medium containing 10% FBS or chemokine medium was added to the lower chamber. Cells were routinely cultured for 16-24 h, at which point the matrix and cells in the upper chamber were wiped with a cotton swab. Cells in the lower chamber were counted under a microscope after crystal violet staining.

### Protein extraction and Western blotting

After the cells were cultured to 90% confluence, the cells were washed twice with precooled PBS and lysed in RIPA lysis buffer (Thermo Fisher Scientific, Waltham, MA, USA) containing 10% protease inhibitor cocktail (Roche, South San Francisco, CA, USA). The protein samples (40 μg each) were separated on 10% polyacrylamide gel by SDS-PAGE and transferred to a PVDF membrane. Then, the membrane was blocked with 5% skim milk at room temperature for 1 hour. The membrane was next incubated with the primary antibody (against LZAP, E-cadherin, p-AKT (s473), AKT or GAPDH) at 4 ℃ for 1 h. The membrane was washed in wash buffer (TBS-T) 3 times for 5 min each and then incubated with an HRP-conjugated secondary antibody (Cell Signaling Technology) at room temperature for 1 h. GAPDH was used as an internal reference. Finally, the film was washed with TBS-T for 30 min, and the bands were visualized using enhanced chemiluminescence (Amersham Corporation, Arlington Heights, IL, USA).

### Subcutaneous tumorigenesis in nude mice

SPF-grade male BALB/c nude mice were purchased from the Zoological Research Institute of the Chinese Academy of Sciences, and all animal-related procedures were performed according to the Animal Protection Committee of Fujian Medical University. Stable high-expressing MGC cells and corresponding control cells (2x10^6) were suspended in 200 ml of phosphate-buffered saline (PBS) and were injected into the right armpit of the nude mice. The tumor volume was measured every 7 days, and the formula was determined by the following equation: (length X width of 2)/2 cm. Mice were sacrificed 35 days after injection, at which point all tumors were excised and weighed.

### Analysis

All experiments were performed at least three times, and results are presented as the average and standard deviation (SD). All the data were processed statistically by SPSS 23.0 software package. The discontinuity data were tested by the χ2 test or the Fisher accuracy test, and the continuity data were tested by t test. Differences were statistically significant at p < 0.05.

## Results

### The expression of LZAP is upregulated in cervical carcinoma

**Figure 1:**
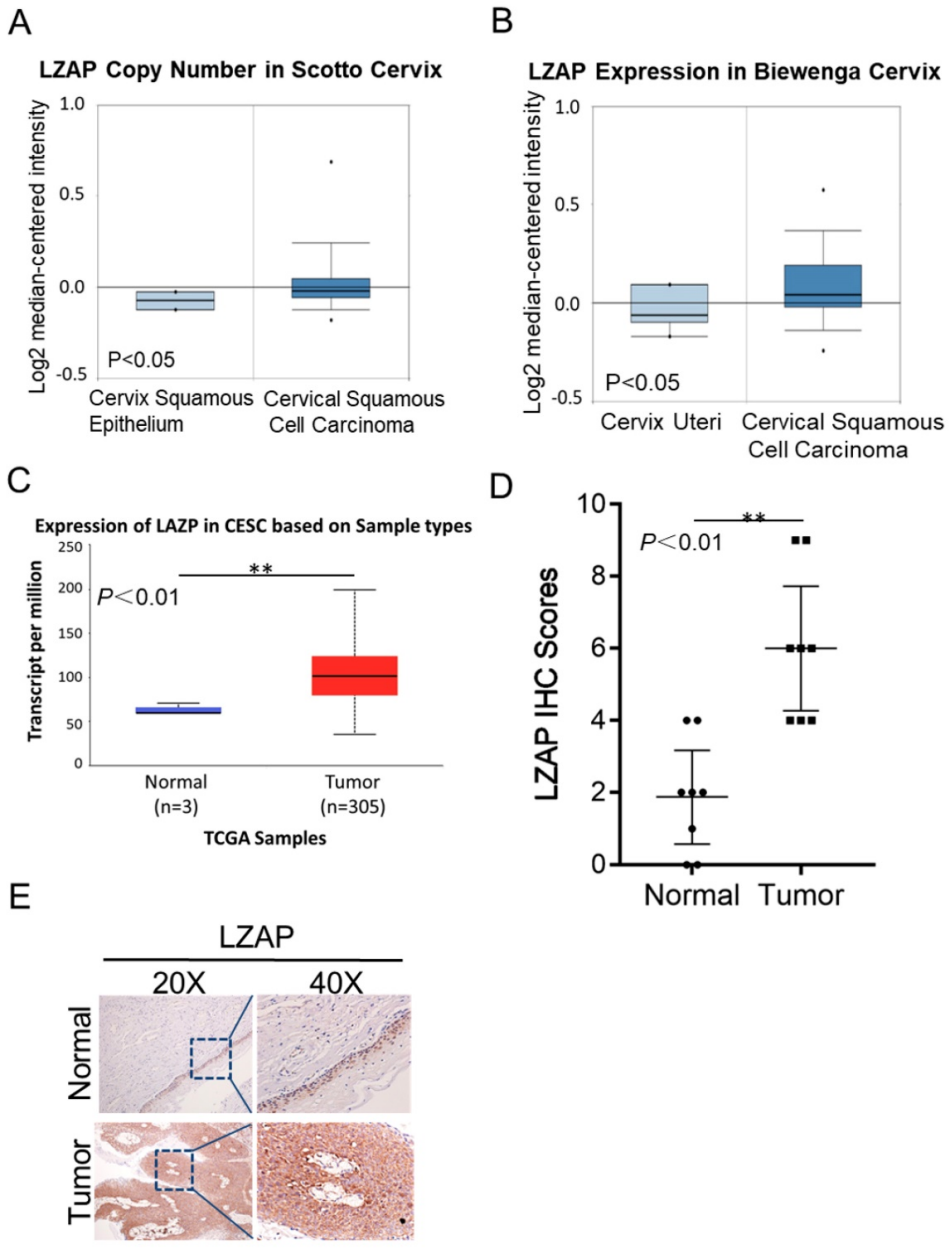
Expression levels of LZAP in cervical carcinoma tissue. (A, B) Oncomine data mining analysis of LZAP levels in the Scotto and Biewenga datasets between normal tissues and cervical carcinoma tissues. (C) Expression of LZAP in cervical squamous cell carcinoma (CESC) according to the TCGA data. (D) The expression of LZAP was significantly elevated in cervical carcinoma tissues compared with adjacent normal tissues. (E) The expression of LZAP protein in cervical carcinoma tissues and corresponding adjacent tissues was analyzed using IHC. (*P < 0.05; **p< 0.01)

To determine the clinical significance of LZAP in patients with cervical cancer, we first performed data mining using the common database Oncomine and analyzed the LZAP expression level. According to the data uploaded by Scotto and Biewenga, the LZAP expression level in cervical cancer tissues was upregulated compared with that in normal tissues (Fig. 1A, B). The results from the TCGA database also showed that the expression of LZAP was upregulated in cervical cancer (Fig. 1C). In addition, we also detected LZAP expression in 8 pairs of cervical cancer tissues and found that the expression of LZAP in cervical cancer tissues was higher than that in corresponding paracancerous tissues (Fig. 1E).

### Overexpression of LZAP promotes the proliferation and metastasis of Cervical Cancer Cells

**Figure 2:**
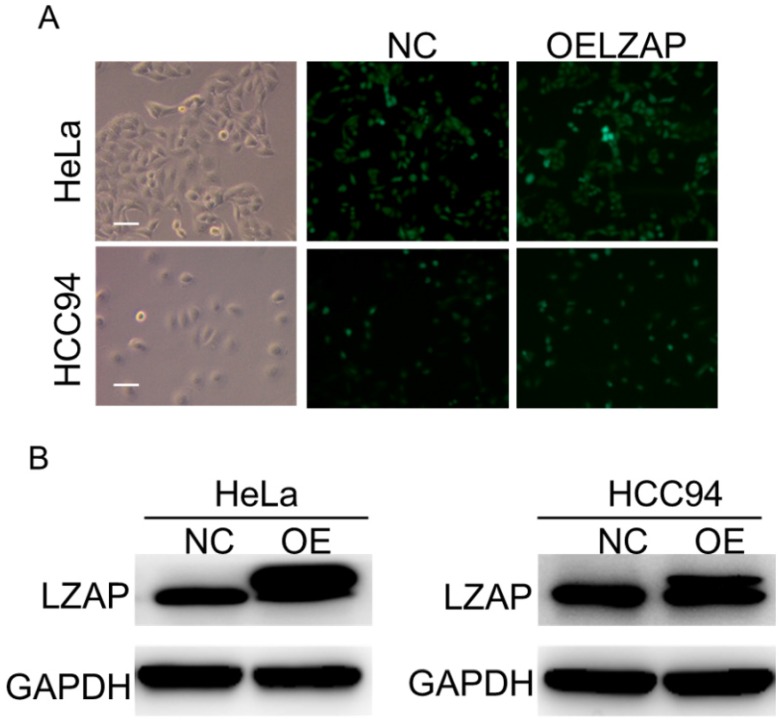
Construction of the overexpressing stable cell line. (A) The overexpression efficiencies of HeLa cells and HCC94 cells after transfection of LZAP. (B) Cell lysates were extracted from HeLa and HCC94 cell lines that stably expressed LZAP protein, which along with GAPDH, was detected by Western blot, scale bar, 50 µm.

**Figure 3:**
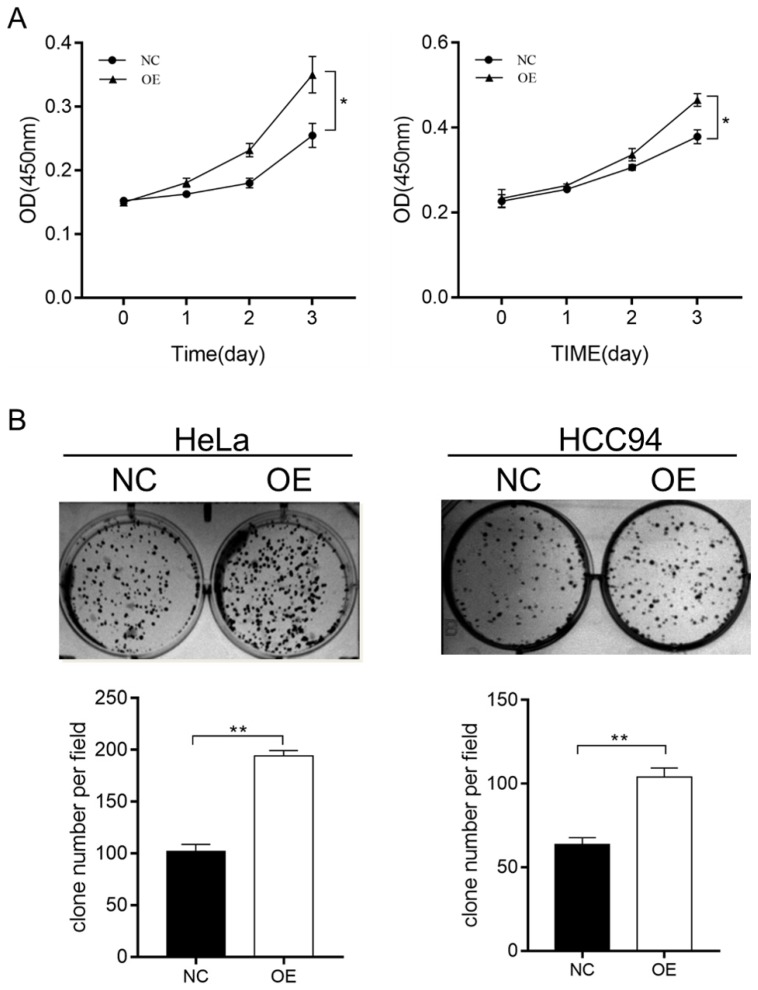
CCK8 and colony-formation assays were used to verify the function of the stably transfected cells. (A) Proliferation of HeLa and HCC94 cell lines stably expressing LZAP was assessed by CCK8 assay. (B) Colony-forming ability of HeLa and HCC94 cell lines stably expressing LZAP was investigated by colony formation assay. (*P < 0.05; **p< 0.01)

**Figure 4:**
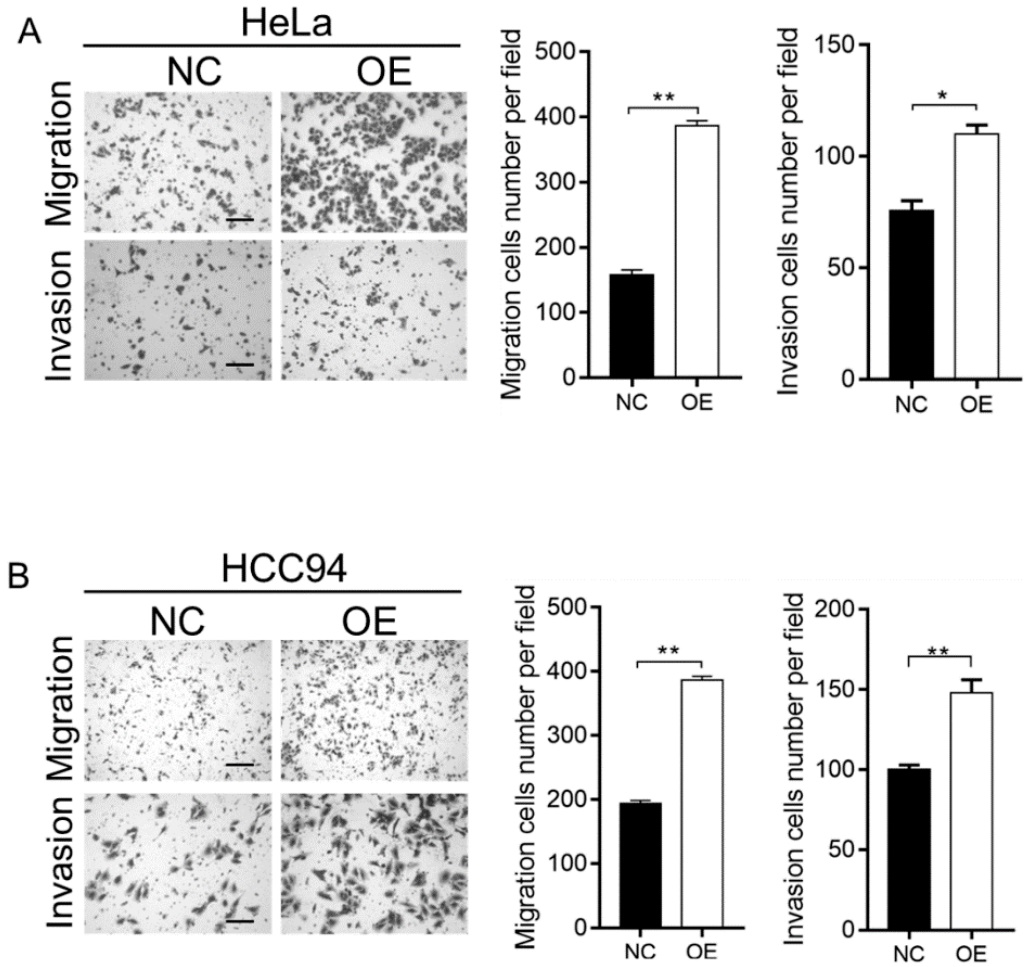
Transwell assay was used to verify the function of the stably transfected cells. (A, B) The migration and invasion abilities of HeLa and HCC94 cell lines stably expressing LZAP was examined using a Transwell assay, scale bar, 50 µm. (*P < 0.05; **p< 0.01)

To further investigate the effect of LZAP on the tumorigenicity of cervical cancer cells, we infected HeLa and HCC94 cervical cancer cells with lentivirus containing the overexpression plasmid (Fig. 2A). The results of the Western blot analysis confirmed that the expression of LZAP in the stable cell line was upregulated (Fig. 2B). The results showed that upregulation of the LZAP gene could promote the proliferation of HeLa and HCC94 cells. According to the CCK8 assay, compared with the negative control group, the proliferation of cervical cancer cells was significantly greater when LZAP was overexpressed (Fig. 3A). Consistent with the above results, the colony formation ability of cervical cancer cell lines after upregulation of LZAP was also significantly higher than that of the negative control group (Fig. 3B). In addition, the invasion assay results showed that overexpression of the LZAP gene could significantly enhance the invasion and migration ability of HeLa and HCC94 cervical cancer cells (Fig. 4A, B). The above experimental results indicate that LZAP can promote the proliferation and metastasis of cervical cancer cells and affect its tumorigenic properties.

### Overexpression of LZAP promotes tumor growth in vivo

**Figure 5:**
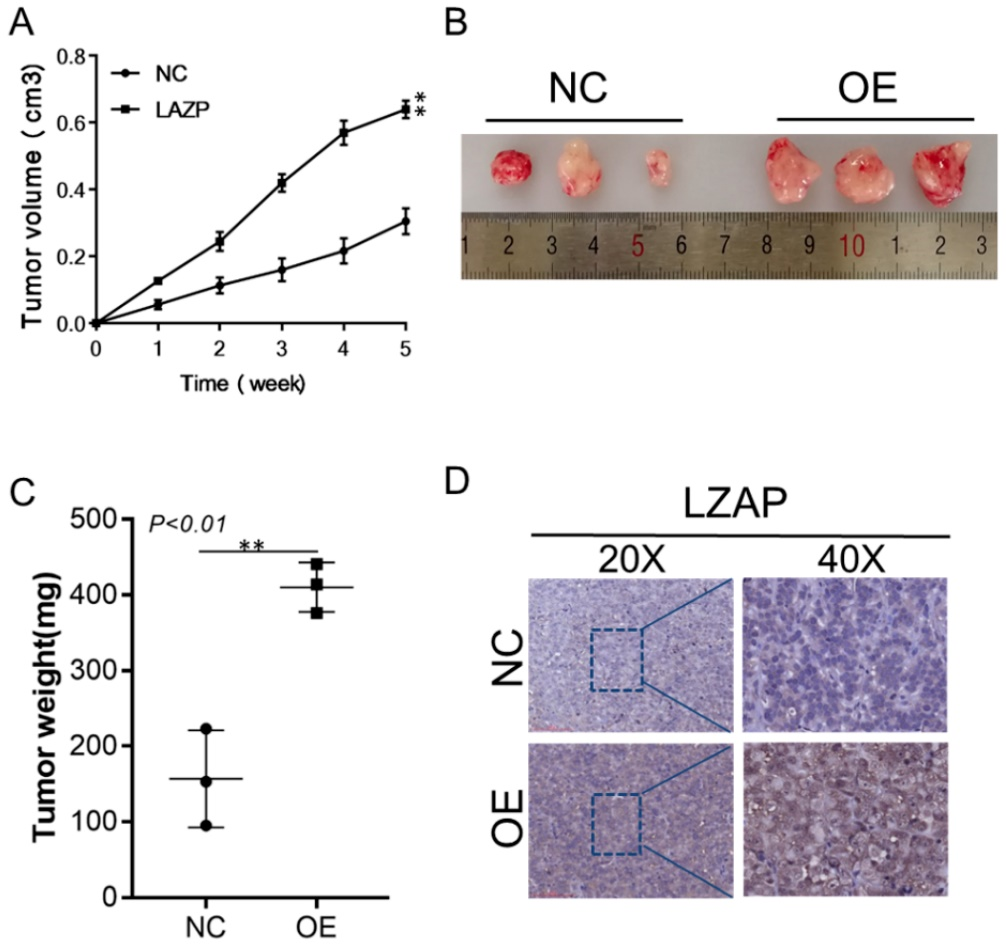
Xenograft models in nude mice. Xenograft models using three sets of stable HeLa cells were established, and the tumors were allowed to grow for 35 days. (A) The size of the xenografts was measured every seven days until the mice were sacrificed. (B) Representative images of the effect of LZAP overexpression are presented. (C) The average tumor weights of the three different groups were compared. (D) The localization and expression of LZAP in xenografts were detected by IHC. (*P < 0.05; **p< 0.01)

The subcutaneous cervical cancer tumor model was established with stable LZAP-overexpressing HeLa cervical cancer cells. The results showed that the size and weight of tumors from the LZAP overexpression group were significantly larger than those of the control group, and this difference was statistically significant (Fig. 5A, B, C). The results also showed that the LZAP protein level in the LZAP overexpression group was higher than that in the control group (Fig. 5D). These results suggest that LZAP can promote the growth of cervical cancer in vivo.

### LZAP promotes the occurrence and development of cervical cancer through AKT and EMT

**Figure 6:**
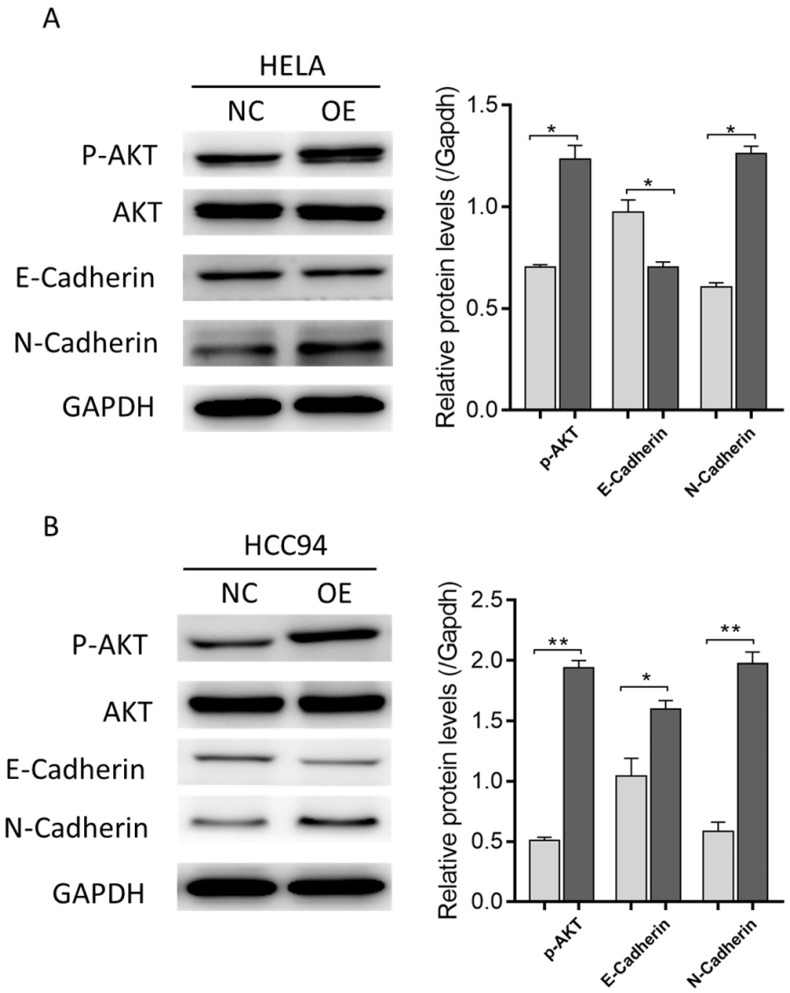
The expression levels of EMT-related proteins indicated by Western blotting in control (NC) and LZAP overexpression groups of HeLa and HCC94 cells. (*P < 0.05; **p< 0.01)

To explore the mechanism by which LZAP affects the tumorigenic properties of cervical cancer, we performed Western blot analysis. The results showed that the overexpression of LZAP promoted the phosphorylation of AKT as well as EMT (Fig. 6A, B), which suggests that LZAP may promote the proliferation and metastasis of cervical cancer cells and the development of cervical cancer by promoting the phosphorylation of AKT.

## Discussion

Tumor development is a complex event involving both environmental and genetic factors. Although the current level of diagnosis and treatment of cervical cancer is increasing, it is still a fatal malignant tumor. It is therefore important to explore the specific biomarkers and effective therapeutic targets of cervical cancer to promote the early diagnosis of this disease and improve the efficacy of molecular targeted drugs.

In recent years, more and more studies have reported that LZAP is involved in the occurrence and development of tumors, but substantial differences have been reported in the expression and role of LZAP in tumors. Mak et al found that LZAP was highly expressed in HCC tissues and that LZAP could promote the metastasis of HCC cells by activating p21-activated protease 4 and downregulating the expression of the tumor inhibitor p14 10. Stavd et al found that the expression level of LZAP was increased in most lung cancer tissues, which played an important role in the diagnosis of lung cancer 11. Wang et al found that the level of LZAP protein was decreased in head and neck squamous cell carcinoma and mediated apoptosis by inhibiting the activation of NF-kB 12. At the same time, Jiang et al also found that LZAP can induce apoptosis by participating in G2/M phase of the cell cycle 13. It has been demonstrated that LZAP plays a different role in different tumor types and participates in multiple classical tumor signaling pathways, which suggests that LZAP may play a key role in tumor development.

In this study, we mined data from the public databases Oncomine and TCGA and analyzed the expression level of LZAP. It was found that the expression level of LZAP in cervical cancer tissues was upregulated compared with normal tissues. To further study the role of LZAP in cervical cancer, we used a lentivirus to construct a stably transfected LZAP-overexpressing cell line. Through in vivo experiments, we observed that overexpression of LZAP can promote the growth of HeLa and HCC94 cervical cancer cells and transplanted tumors in nude mice. This suggests that LZAP can promote the proliferation of cervical cancer cells. The Western blot results showed that overexpression of LZAP could increase the phosphorylation level of AKT at position 473. AKT is widely expressed in tissues and is the primary downstream target of the PI3K/AKT pathway 14, 15. As the central link of multiple cell signaling networks, AKT plays an important role in regulating the processes of cell proliferation, differentiation and survival 16. It was found that AKT could upregulate the expression of the tumor inhibitor c-Myc, regulate the cell cycle and promote cell proliferation. Moreover, cell lines stably transfected with LZAP could induce epithelial stroma transformation, downregulate the expression of E-cadherin, decrease cell adhesion, and promote the invasiveness and metastasis of cells, and thus this protein plays an important role in the occurrence and development of tumors 17.

In addition, the results of the invasion assay showed that overexpression of LZAP could promote the invasiveness and migration of HeLa and HCC94 cervical cancer cells. Western blot also showed that the overexpression of LZAP downregulated the expression of E-cadherin protein and upregulated the expression of N-cadherin, which promoted the EMT process. EMT refers to the transdifferentiation of epithelial cells into mesenchymal cells under normal physiological and specific pathological conditions 18. EMT is a complex process characterized by the loss of polarity, adhesion and stroma in epithelial cells. Increasing evidence suggests that EMT plays a key role in the metastasis of a variety of cancers, mainly by reducing the expression of epithelial markers and increasing the expression of stromal markers, which reduces cell adhesion and increases cell migration, thus increasing the capacity for invasion and cell metastasis 19, 20. During the process of EMT, an important molecular event is the abnormal expression of cadherins, in which E-cadherin and N-cadherin are the two types most closely related to tumor metastasis 21. Wheelock et al presented the concept of "cadherin conversion" (cadherin switch), which is an important mechanism of EMT 22. Other scholars have intensively studied the classical cadherin molecules and found that the deletion or mutation of E-/N-cadherin molecules is closely related to the occurrence and metastasis of many tumor types such as liver cancer, gastric cancer, breast cancer and lung cancer 23, 24.

In summary, the current study shows that upregulation of LZAP can promote the phosphorylation of AKT and can enhance the EMT-induced invasion and migration abilities of cervical cancer cells, which promotes the proliferation of cervical cancer cells and disease progression. LZAP is an oncogene in cervical cancer, which suggests that LZAP may be a potential new index of cervical cancer treatment, but this requires confirmation by further large-scale clinical studies. Furthermore, we found that the LZAP is targeted at AKT and EMT. In some studies, there were found that PI3K/AKT/GSK-3βpathway which can promote the epithelial-to-mesenchymal transition 25, 26. However, how LZAP regulates AKT phosphorylation and the occurrence of EMT is the limitation of this study, which requires further investigation.
